# Exploring plasmonic coupling in hole-cap arrays

**DOI:** 10.3762/bjnano.6.1

**Published:** 2015-01-02

**Authors:** Thomas M Schmidt, Maj Frederiksen, Vladimir Bochenkov, Duncan S Sutherland

**Affiliations:** 1 Interdisciplinary Nanoscience Center (iNANO), Aarhus University, Gustav Wieds Vej 14, 8000 Aarhus, Denmark; 2 Department of Chemistry, Lomonosov Moscow State University, Moscow, Russian Federation

**Keywords:** caps, colloidal lithography, hybridization, localized surface plasmon resonance, near field, SRO hole arrays

## Abstract

The plasmonic coupling between gold caps and holes in thin films was investigated experimentally and through finite-difference time-domain (FDTD) calculations. Sparse colloidal lithography combined with a novel thermal treatment was used to control the vertical spacing between caps and hole arrays and compared to separated arrays of holes or caps. Optical spectroscopy and FDTD simulations reveal strong coupling between the gold caps and both Bloch Wave-surface plasmon polariton (BW-SPP) modes and localized surface plasmon resonance (LSPR)-type resonances in hole arrays when they are in close proximity. The interesting and complex coupling between caps and hole arrays reveals the details of the field distribution for these simple to fabricate structures.

## Introduction

The interaction of metal nanostructures, both highly symmetric and asymmetric, with light has been investigated extensively in the last years. Localized surface plasmon resonances (LSPR’s) at these structures provide a wealth of interesting phenomena with a broad range of proposed applications [1]. Structures have been fabricated by both chemical synthesis, e.g., nanoshells [2], nanoeggs [3], nanorice [4] and lithography, e.g., nanodiscs [5], nanorings [6] to nanocaps [7] giving different degrees of ease of fabrication and control of shape, relative position and orientation of structures. Nanocaps have attracted interest by combining simple production [8] and both unique properties such as bending of light [9] resulting from reduced symmetry compared to nanoshells and other more common plasmonic effects such as enhanced local electromagnetic fields [8]. Another related plasmonic structure of interest relates to nanocavities either as isolated structures such as nanoholes in metallic thin films [10] or as interconnected nanocavities in a metallic matrix [11]. LSPR’s at cavities and nanoholes can couple to the propagating surface plasmon polariton (SPP) modes as well as generate the high local electromagnetic fields. These enhanced local electromagnetic fields of the different plasmonic structures have been applied to enhance optical transitions such as in Raman spectroscopy [12] (as surface enhanced Raman scattering – SERS) and fluorescence [13] (as surface enhanced fluorescence – SEF) where the strong electromagnetic fields can greatly enhance excitation and emission. Other uses of the enhanced local electromagnetic fields are as nanoscale lenses to carry out high resolution near field optical lithography [14], as antennas [15] for directing light emission at the nanoscale or increasing interaction cross-sections for example for light harvesting applications [16].

Interactions between resonances at plasmonic nanostructures in close proximity can significantly alter their optical properties and give spectral tuneability and higher localized fields. These highly interesting interactions can be explained by the hybridization model which describes the complex plasmonic interactions as a combination of elementary plasmon modes [17]. Good examples of this are seen in the cases of nanorice [4], nanostars [18], semishells [8] and nanoparticle dimers [19–20]. Such hybridized systems have shown promise for enhancement of plasmonic sensing systems [2,4,21]. Nanocap-hole arrays are extremely simple coupled structures to produce based on colloidal lithography with the potential for use in sensing applications. They have recently been applied for SERS enhancement [22].

Here we focus on investigating the optical properties of short range ordered arrays of nanocap-holes coupled structures and interpret them in terms of hybridization of their more elementary components. We fabricate these structures utilizing colloidal monolayer masks as a template and compare experimental extinction data to finite-difference time-domain (FDTD) simulations. We show strong coupling of the dipolar and quadrupolar nanocap resonances with the Bloch wave-SPP (BW-SPP) and LSPR type hole array resonances.

## Experimental

### Nanostructure design

**Figure 1:**
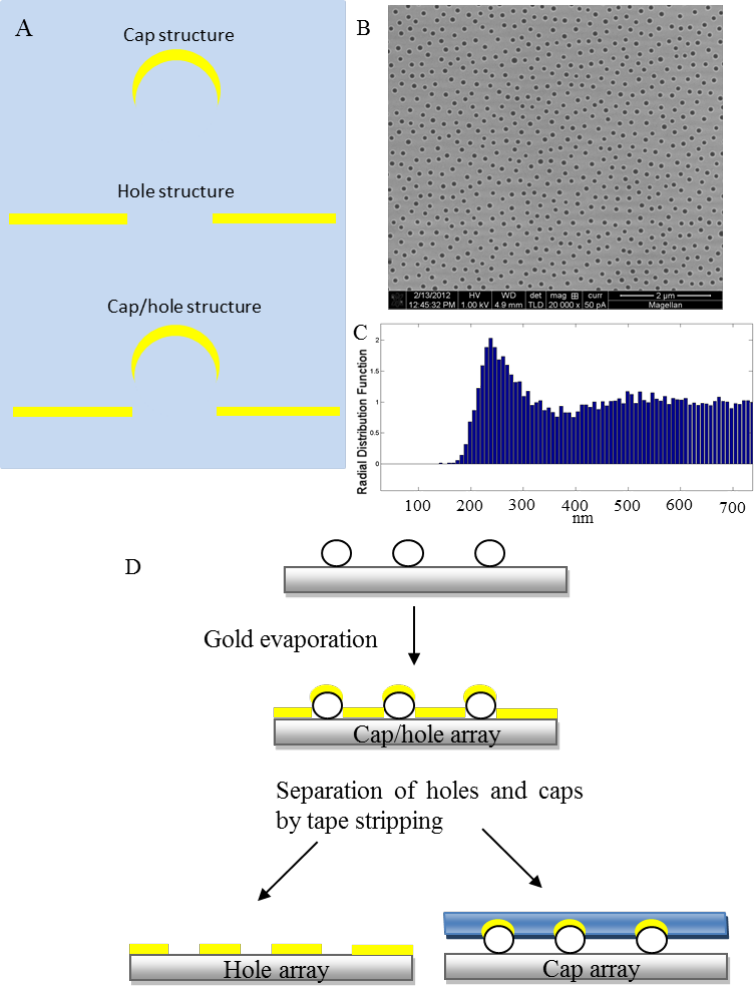
A) Schematic of the design of the plasmonic gold structures fabricated by colloidal lithography. B) SEM image showing the distribution of a 110 nm hole array in 20 nm thin Au film after removal of the gold-caps by tape stripping. C) Radial distribution function showing the characteristic average spacing between holes of the same hole array. D) Fabrication route for the plasmonic structures.

Figure 1a shows a schematic of the design of the plasmonic gold structures fabricated by colloidal lithography: Cap structure, hole structure and cap/hole structure. The fabrication route can be seen on the schematic in Figure 1d. The common mask system for fabricating all the structures means that they have the same distribution and orientation as well as similar geometric features. Due to the needs of the fabrication process the gold structures are in contact with the polystyrene (PS) particle, glass substrate and/or the clean room tape which modify the local dielectric environment of the nanoparticle and have to be taken into account when understanding the spectral features.

### Materials

White sulfate latex polystyrene particles with diameter of 0.11 µm were obtained from Invitrogen Denmark. Deionized water with 18.2 MΩ resistivity from a Millipore Milli-Q water system. Poly(methyl methacrylate) (PMMA) (*M*_w_ 120,000), Polystyrene (*M*_w_ 280,000), PDDA (poly(diallydimethylammonium chloride)) (*M*_w_ 200,000–350,000), PSS (poly(sodium 4-styrenesulfonate)) (*M*_w_ 70,000) and toluene were obtained from Aldrich Denmark. Poly aluminum chlorohydrate (PAX XL 60) was obtained from Kemwater Denmark. Round glass cover slides of soda-lime glass # 3 from Menzel–Gläser (*d* = 24 mm) Germany. Silicon wafers (thickness of 356–406 µm with a diameter of 76.2 ± 0.63 mm), pre-cut about half way through for controlled breaking from SI-MAT Germany.

### Sample preparation

Sample preparations were done as previously described by Schmidt et al. [23]. The round glass cover slide or silicon wafer substrates were cleaned by sequential ultrasonic agitation for 10 min in acetone, ethanol and deionized water followed by drying under N_2_ flow. Just before fabrication the surface was cleaned from any residual organic contaminated by a 20 min UV/ozone treatment in a purpose built machine with the sample placed ≈10 mm from a 100 W mercury grid lamp from BHK Inc. Colloidal assembly of the nanoparticles to generate dispersed colloidal monolayers masks was carried out by an approach described elsewhere [24]. In brief the surfaces were modified with a polyelectrolyte triple layer by sequential deposition from a 2% w/v PDDA, a 2% w/v PSS and a 5% w/v PAX XL 60 all in deionized water solution. Each layer was deposited by sequential exposure of the surface to the solution for 30 s, rinsing in deionized water for 30 s and dried under N_2_ flow.

The assembly of the colloidal particles onto the triple layer modified substrates was then carried out by exposure of the dry substrates to a 0.2% solution of polystyrene particles in deionized water for 120 s followed by rinsing in deionized water for 60 s and careful drying under N_2_ flow. Care was taken to dry quickly and to prevent rewetting of the surface. The assembled colloidal monolayer masks were modified by evaporation of a thin film of gold (between 10–40 nm in thickness) utilizing a homebuilt system for Electron Beam stimulated Physical Vapor Deposition, EB-PVD, (2 kV e-gun, base pressure 1 × 10^−7^ Torr, deposition rate of 0.1–0.4 nm/s) to generate cap/hole arrays. The cap and hole arrays were generated in the same process and were separated physically by tape striping with a transparent adhesive tape (90 µm thick blue PVC Tape from Semiconductor Production Systems). The tape was subsequently placed onto a glass substrate for characterization. The tape stripped cap/hole arrays left behind hole arrays.

### Characterization

The optical spectra were measured using a UV-3600 UV–vis–NIR spectrophotometer from Shimadzu with a wavelength range of 185–3300 nm in dual beam mode. A spectral resolution of 5.0 nm and a sampling interval of 1.0 nm were used. An area of around 10 mm high and 3 mm wide was probed. AFM images were taken with an atomic force microscopy (AFM) from Multimode with Veeco nanoscope 5 used in tapping mode. Olympus OMCL-TR400PSA-2 cantilevers with a resonant frequency between 11 and 34 kHz and a tip radius of less than 20 nm were used. The SEM images were taken with a Nova 600 NanoLab XHR Magellan scanning electron microscope (SEM) from FEI generally with energies of 1–5 kV and nominal spot size ≈1 nm.

Computer simulations of extinction spectra and charge/field plots analysis were carried out using the finite-difference time-domain method (FDTD Solutions, Lumerical). The gold material was modeled using the fit to experimentally measured dielectric data [25]. Constant refractive index values *n* = 1.58 and *n* = 1.52 were used for polystyrene and glass, respectively. Hole arrays were simulated on glass in air, the combined arrays were simulated on glass with the caps on top of polystyrene spheres 110 nm in diameter. For closer spacing of cap and hole arrays the polystyrene spheres were truncated at the glass interface. The caps were simulated in air. The non-uniform mesh was applied to the whole simulation box and a uniformly fine-meshed region with the grid size of 1.5 nm was used to reproduce the curved structures and avoid the sharp edges. For all the simulations the direction of the light propagation is normal to the surface and the polarization horizontal in the figure plane as in the experiment. The charge and field plots were analyzed for the resonance frequencies, obtained from simulated spectra.

## Results and Discussion

The colloidal lithography approach enables the simple and robust fabrication of short range ordered (SRO) arrays of nanostructures [5,26–28]. Figure 1b shows the distribution of a hole array after removal of the capped particles by tape stripping. The well separated short range ordered array is resulting from the electrostatic repulsion between already adsorbed particles and later arriving ones during the particle deposition step of the colloidal lithography fabrication. The radial distribution function of the holes can be seen in Figure 1c. SRO hole arrays fabricated by this approach have been studied in terms of their optical response and been used in sensing applications extensively in recent years [29–34]. The arrays of structures of the different sample types studied here are made from the same specific colloidal mask pattern giving them identical distributions, see Figure 1d. The structures have a characteristic center to center spacing around 220–250 nm and average diameter of 110 nm with a standard deviation of 5 nm.

**Figure 2:**
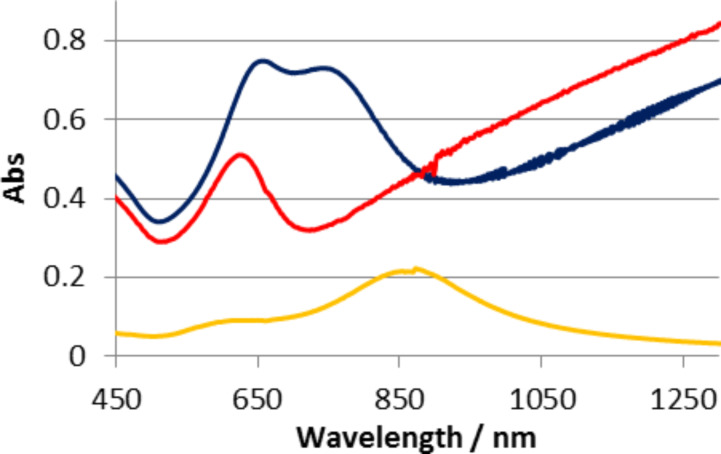
Extinction spectra of arrays of cap (yellow), hole (red) and cap/hole (blue) structures with 110 nm diameter in 20 nm gold on glass substrates.

**Figure 3:**
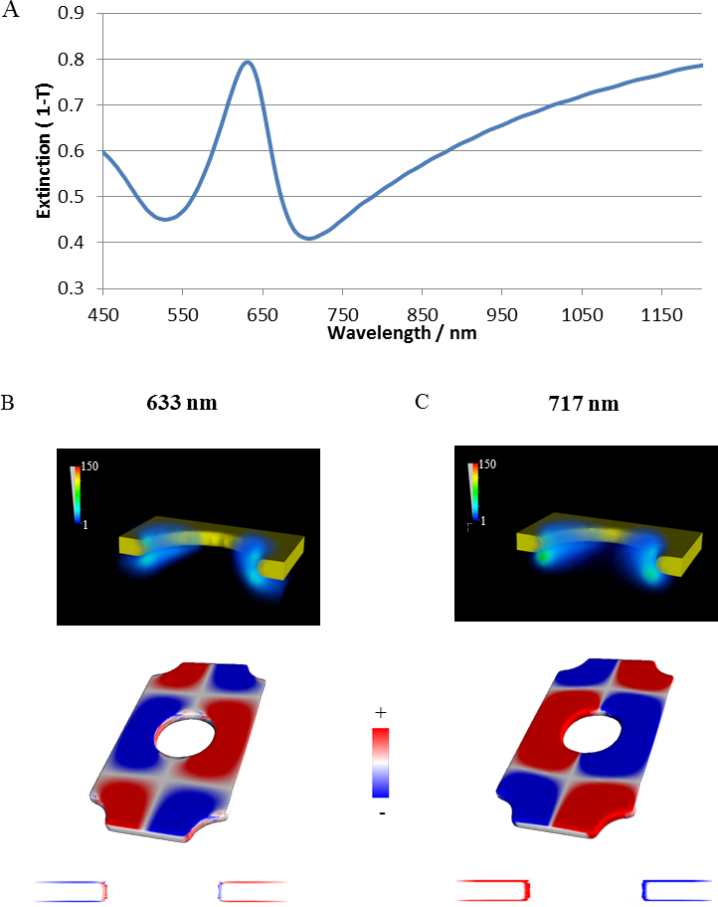
A) Simulated extinction spectra of a hole array (holes diameter 110 nm) in 20 nm thick gold film. Underneath the E-field intensity (|*E*|^2^/|*E*_0_|^2^) and charge density plots for the peak at 633 nm (B) and dip at 717 nm (C) is shown. The direction of the light propagation is normal to the surface and the polarization horizontal in the figure plane.

**Figure 4:**
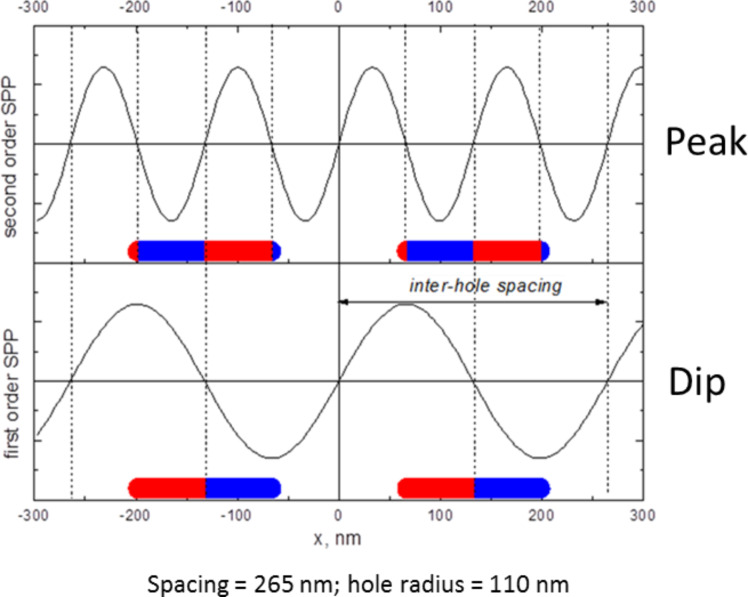
Schematic view of the first order and second order BW-SPP’s charge oscillations for the hole arrays.

We explore the plasmonic resonances of SRO hole arrays, cap arrays and coupling between cap and hole arrays by UV–vis extinction spectroscopy. Figure 2 shows experimental extinction spectra of arrays of holes, arrays of caps or of coupled Cap/hole arrays schematically shown in Figure 1a formed from dispersed monolayers of 107 nm colloids with Au film thickness of 20 nm. The cap array and hole array are generated by physically separating the coupled cap/hole arrays by tape striping and thus show the extinction spectra of the specific cap and hole arrays used in the cap/hole arrays. The coupled cap/hole arrays cannot be explained by a simple addition of the two component spectra (even taking into account that the cap array is red shifted by the higher refractive environment resulting from the tape used for striping) suggesting that coupling is occurring. We first explore and understand the origin of the component spectra. The hole array shows a resonant peak that can be seen at 620 nm and a dip at around 700 nm. The exact origin of these two spectral features has been debated in recent years [29,33–35] and what has emerged is that coupling of SPP-modes with LSPR modes appears to be occurring. Sannomiya et al. [33] have assigned the peak as a collective BW-SPP type mode that can be excited by the incoming EM-field and the dip as a more localized resonance with the holes comparable to LSPR’s in metal disks or other cavities [11]. Recent work has focused on refractive index sensitivity increases observed when restructuring the substrate around the holes and have suggested that the both the BW-SPP and LSPR type assigned resonances have considerable localized character [34]. Here we have carried out FDTD simulations of holes on glass substrates as hexagonally close packed periodic arrays in a similar way to previous work [33] and plot the field and charge distributions in 3D in an effort to understand the origin of these two resonances (the peak and dip seen experimental in Figure 2 and via simulations in Figure 3a). So we have experimental SRO arrays but the optics are described by simulations of periodic arrays. The field plots for the dip position (Figure 3c) shows a clear dipole resonance localized at the contact point between the edge of the hole and the substrate. The charge plots for this resonance (Figure 3c) show that the charge within the edge of the hole reflects the charge of the surrounding upper and lower gold surfaces which resembles a first order SPP mode. The localized field characteristics and the extended charges on the surface imply a coupling between an SPP mode and a more localized mode. It can be regarded as an in phase coupling of a dipolar cavity mode with the first order SPP Bloch wave mode. The redistribution of the field to the substrate side of the metal film is expected from the high refractive index substrate and has important ramifications for the refractive index sensitivity of the structures [36]. The picture for the higher energy (peak) position is more complex. The field shows again a high magnitude at the edge of the hole with a redistribution to the substrate side (down in Figure 3b) but clearly less localized to the hole itself than the field plots for the dip. The charge plots surprisingly show a different phase at the hole wall compared to the surrounding metal surfaces. We note that although FDTD calculations similar to these have been carried out before, apparently the charge distributions have not been plotted previously. The charge distributions can be understood to arise from a BW-SPP wave of higher order as shown in Figure 4. The frequency of a second order BW-SPP wave would not coincide with that of any dipolar hole LSPR resonance. The local field distribution arises then from the shape of the metal surface rather than a localized resonance. The excitation of different order SPP modes directly via holes in metal films (as opposed to indirectly via coupling to LSPR resonances at holes) was suggested from theoretical approaches [35]. Here we clarify the assignment of the BW-SPP modes in our system (which is typical of the systems used currently [33–34] in literature) as a second order SPP mode, with the localized distribution of the field arising from the abrupt presence of a hole in the metal film rather than an LSPR type character. We assign the dip to be a bonding hybridization of dipolar LSPR modes at the holes and a first order SPP mode. These assignments are consistent with the recent experimental and theoretical reports [30,33].

**Figure 5:**
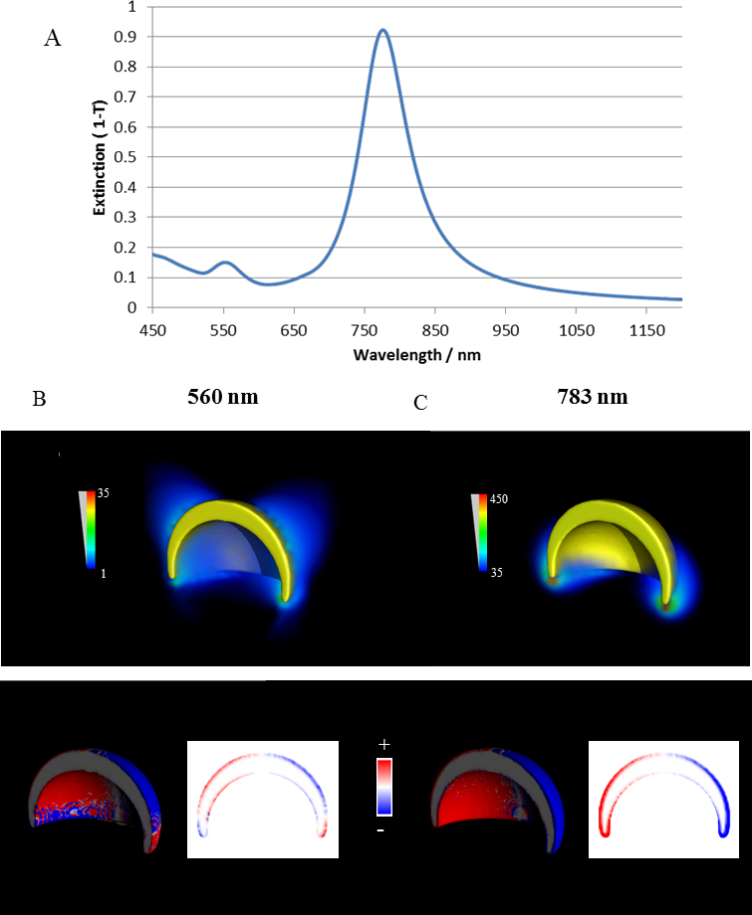
A) Simulated extinction spectra of a 20 nm thick gold cap (110 nm in diameter). Underneath the E-field intensity (|*E*|^2^/|*E*_0_|^2^) and charge density plots for the peaks at 560 nm (B) and 783 nm (C) are shown. The direction of the light propagation is normal to the surface and the polarization horizontal in the figure plane.

The cap array shows a main LSPR peak at 860 nm and a minor LSPR peak at approximately 610 nm. This spectral profile has previously been identified as corresponding to dipolar and quadrupolar modes respectively [37]. FDTD simulations of caps were carried out with caps immersed in air and simulated extinction spectra are shown in Figure 5a. The forms of the experimental spectra are reproduced identifying the two resonances: a low energy resonance with a relatively high intensity and a high energy but low intensity resonance. E-field intensity (|*E*|^2^/|*E*_0_|^2^) and charge density plots (shown in Figure 5b) confirm the low energy resonance as a dipolar mode and the high energy resonance as a quadrupolar mode. This is in accordance with previous studies of semi-shells [37–38]. The spectral position of the experimental cap resonances are red shifted by the higher refractive index of the polymeric tape.

**Figure 6:**
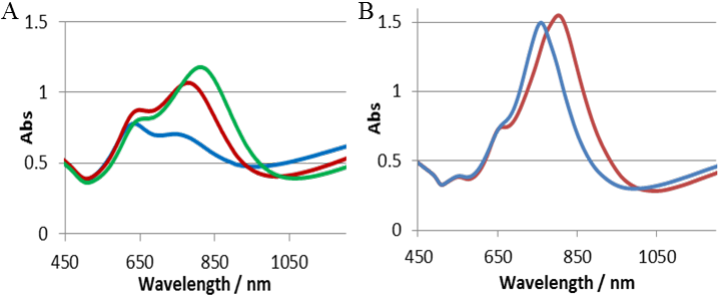
A) Extinction spectra of 110 nm PS particle gold cap/hole array with a gold film thickness of 30 nm where samples were not heated (blue) or post-heated to 116 °C (red) or 125 °C (green). B) Simulations of the non-heated structure in 6a (Blue) and with the cap lowered 10 nm (red).

The extinction spectrum of as-prepared cap/hole array in Figure 6a (blue) contains two LSPR peaks one at 650 nm and one at 740 nm. We have experimentally explored the existence of coupling between the cap and hole arrays by a novel approach to vary the vertical spacing between the arrays. We make use of the glassy properties of the templating polystyrene colloids by heating the particles to temperatures close to and above the glass transition of polystyrene to allow reshaping of the colloid giving increased wetting of the silica surface and thereby reducing the vertical distance between the caps and holes. Any coupling between caps and hole arrays should be strongly distance dependent. Figure 6a shows experimental spectra of cap/hole arrays after no thermal treatment (blue) or heating to 116 °C (red) or 125 °C (green) respectively (the temperatures should be compared to the glass transition of bulk PS (ca. 106–110 °C). AFM measurements confirmed that the height of the caps relative to the gold surface is reduced from ≈120 nm to 85 nm hole arrays (see Supporting Information File 1, Figure S1) as a result of reshaping of the colloids with the highest temperature implying that the gap between the lower rim of the caps and the upper edge of the hole array is <10 nm. The extinction spectra reveal a systematic red shift in the low energy peak for the higher temperature thermal treatments. While the reshaping of the PS particle can have resulted in a change of the dielectric environment around the cap and resulted in some shift in the position of the resonance peaks purely from a refractive index sensitivity origin, the magnitude of the change in the spectral profile with decreasing gap between the caps and hole arrays strongly supports the idea of coupling between them.

**Figure 7:**
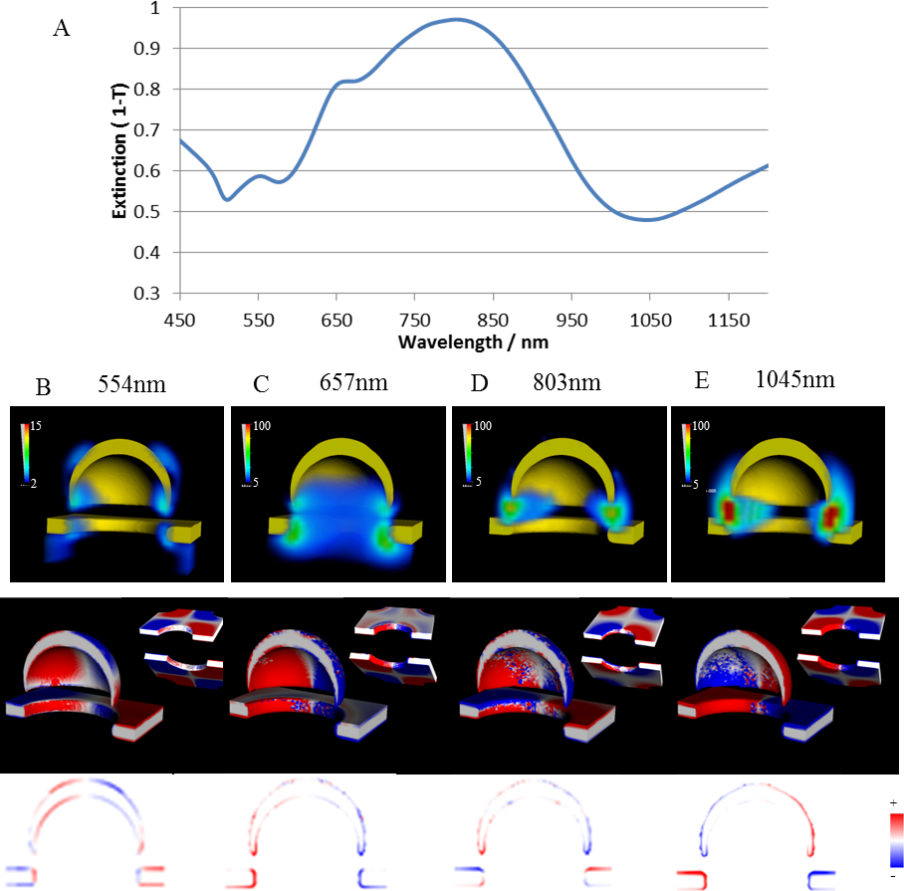
A) Simulated extinction spectra of a 20 nm thick cap/hole structure (diameter 110 nm). Underneath the E-field intensity (|*E*|^2^/|*E*_0_|^2^) and charge density plots for the peaks at 554 nm (B), 657 nm (C), 803 nm (D) and dip at 1045 nm (E) are shown. In the 3D charge density plots insets of the hole parts without the cap are shown from the bottom and the top for easier presentation of the charges at the gold film surface. The cap was still taken into account for these plots. The direction of the light propagation is normal to the surface and the polarization horizontal in the figure plane.

**Figure 8:**
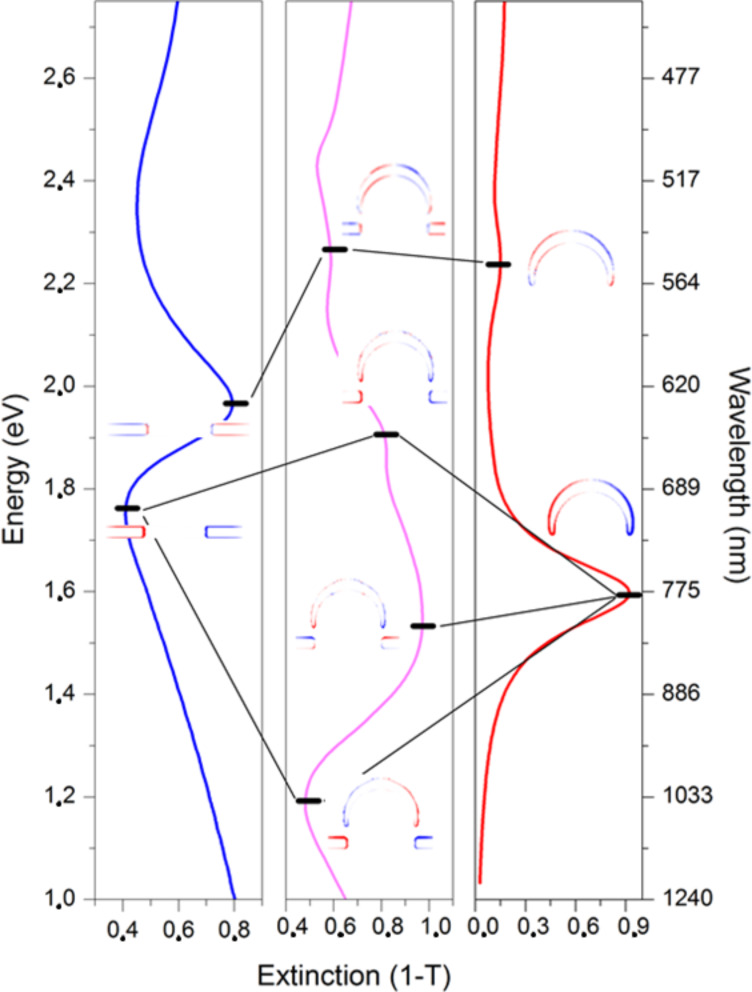
Hybridization diagram of the gold cap/hole structures showing the coupling between the hole and cap modes.

To visualize the plasmonic modes of cap/hole array structures FDTD calculations of the combination of the SRO hole arrays (seen in Figure 3) and caps in appropriate refractive index were made. Calculated extinction spectra are shown in Figure 6b for a distance of 55 nm (blue) and 45 nm (red) between the edge of a cap and the top surface of the gold film, respectively. The form of the spectra with the red shift of the dominant peak and associated peak splitting when reducing the distance between the cap and hole arrays is reproduced. A higher energy peak (554 nm) observed in the calculated spectra is clearly defined which is present in the experimental spectral only as a weak shoulder. The field and charge plots for the closest separation configuration are shown in Figure 7b–e for the three peaks seen in the calculated spectra (554 nm, 657 nm, 803 nm) as well as the dip at long wavelength (1045 nm). The charge plots reveal that the highest energy resonance results from the coupling between a quadrupolar cap resonance and the BW-SPP (higher energy) hole resonance. The charges at the lower edge of the cap are in phase with the charges on the inside of the hole which apparently leads to a shift to higher energy by electrostatic destabilization. The three lower energy resonances (the two peaks and the low energy dip) result from coupling between the dipolar cap resonance and hole resonances. The peak at 657 nm and the dip at 1045 nm can be understood in terms of in phase (anti-bonding) and out of phase (bonding) coupling respectively between the dipolar cap peak and localized hole resonance (seen as the lower energy dip in the hole spectra (Figure 3a and identified as a coupling between a dipolar LSPR cavity mode and the first order BW-SPP). The bonding lower energy resonance (dip at 1045 nm) results from a shift to lower energy driven by a simple electrostatic stabilization. The anti-bonding higher energy resonance (peak at 657 nm) results from a shift to higher energy resulting from electrostatic destabilization around between the hole edge and the lower rim of the cap. The elemental hole plasmon is largely shifted to the lower surface of the metal as a result of the high dielectric substrate. The anti-bonding higher energy peak shows the same field distribution as the elemental hole resonance, while the electrostatic stabilization in the bonding mode leads to a redistribution of the field to the gap between the upper edge of the hole and the lower rim of the cap. The broad and strong resonance at 803 nm interestingly shows a different phase at the upper and lower metal surfaces which indicates a role for the dark anti-symmetric SPP mode (here revealed as an anti-symmetric BW-SPP mode by coupling to the bright dipolar cap mode). The significant coupling to the far field for this mode results from the large structural asymmetry between the cap and the BW-SPP. The field distributions, particularly for the two anti-symmetric modes (peak at 803 nm and dip at 1045 nm) show a strong field confinement between the cap and the hole with field enhancement up to 25 and 29. Figure 8 shows the coupling schemes schematically superimposed on the calculated extinction spectra for the elementary cap and hole spectra (right and left respectively) and the coupled system (central).

## Conclusion

We have studied the coupling between elementary cap and hole resonances. The cap/hole system is easy to produce and here we present a novel method to increase the coupling between cap arrays and hole arrays utilizing the glass transition of polystyrene colloidal particles used as a templating mask. The strong coupling results in localization of high field sites between the cap and the upper edges of the holes which has been used in recent work to enhance optical processes [22] . We provide a detailed description of the modes, interestingly identifying bonding between the caps and both bright symmetric and dark antisymmetric SPP modes. The coupling of LSPR modes to dark anti-symmetric SPP modes has not to our knowledge been previously reported. The coupling present in this simple to fabricate system can be used both to study dark SPP modes and/or for rational design of sensors through plasmon enhancement of optical processes (e.g., SERS or SEF) and/or engineering of the near field (lifting the SPP modes out of the substrate) for refractive index sensing.

## Supporting Information

File 1 AFM measurements of the height from the gold film surface to the gold cap on the top the PS particle.
