# Supplementary material for: Exploring plasmonic coupling in hole-cap arrays
Source: Beilstein J Nanotechnol. 2015 Jan 2;6:1–10. doi: 10.3762/bjnano.6.1 (PMC4311723; doi:10.3762/bjnano.6.1)
Supplement: File 1 — AFM measurements of the height from the gold film surface to the gold cap on the top the PS particle. [file Beilstein_J_Nanotechnol-06-01-s001.pdf]

Supporting Information

for

Exploring plasmonic coupling in hole-cap arrays

Thomas M. Schmidt[1], Maj Frederiksen[1], Vladimir Bochenkov[1,2] and Duncan S. Sutherland[*1,§]

Address: [1]Interdisciplinary Nanoscience Center (iNANO), Aarhus University, Gustav Wieds Vej 14, 8000 Aarhus, Denmark and [2]Department of Chemistry, Lomonosov Moscow State University, Moscow, Russian Federation

Email: Duncan S. Sutherland - duncan@inano.au.dk

* Corresponding author

[§] Tel: +45 23 38 57 89

AFM measurements of the height from the gold film surface to the gold cap on the top the PS particle

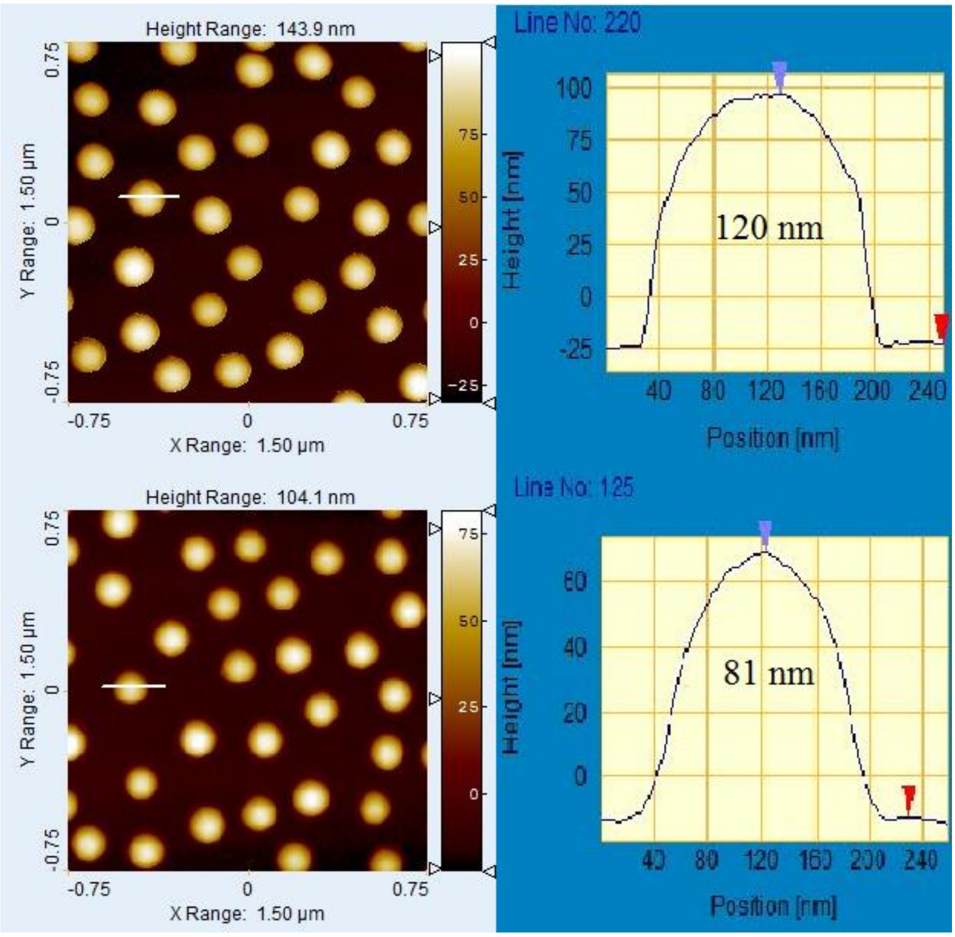

Figure S1: AFM measurements of the height from the gold film surface to the gold cap on the top the PS particle (To the right a single line scan is shown). Upper picture: No heating. Lower picture: Heated to 125 °C.
